# Self-assembly of gold supraparticles with crystallographically aligned and strongly coupled nanoparticle building blocks for SERS and photothermal therapy†
†Electronic supplementary information (ESI) available: Fig. S1–S13. See DOI: 10.1039/c6sc02465c


**DOI:** 10.1039/c6sc02465c

**Published:** 2016-06-20

**Authors:** S. Paterson, S. A. Thompson, J. Gracie, A. W. Wark, R. de la Rica

**Affiliations:** a Department of Pure and Applied Chemistry , WestCHEM , University of Strathclyde , Technology and Innovation Centre , 99 George Street , Glasgow , G1 1RD , Scotland , UK . Email: roberto.delarica@strath.ac.uk; b Department of Chemistry and Biochemistry , Hunter College-City University of New York , New York 10065 , USA

## Abstract

A new method is introduced for self-assembling citrate-capped gold nanoparticles into supraparticles with crystallographically aligned building blocks. It consists in confining gld nanoparticles inside a cellulose acetate membrane. The constituent nanoparticles are in close contact in the superstructure, and therefore generate hot spots leading to intense Surface-Enhanced Raman Scattering (SERS) signals. They also generate more plasmonic heat than the nanoparticle building blocks. The supraparticles are internalized by cells and show low cytotoxicity, but can kill cancer cells when irradiated with a laser. This, along with the improved plasmonic properties arising from their assembly, makes the gold supraparticles promising materials for applications in bioimaging and nanomedicine.

## Abstract 2

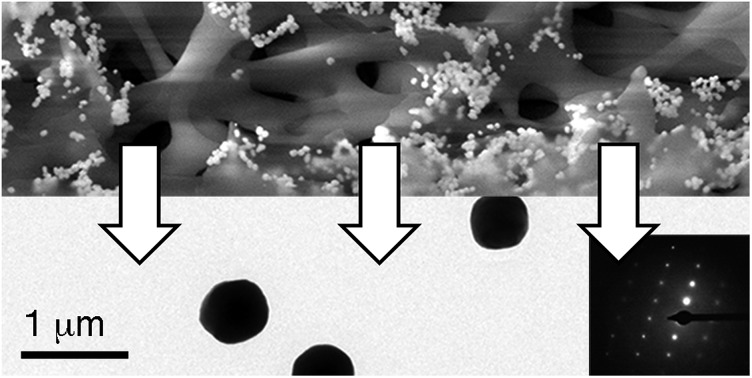
A new method is introduced for self-assembling citrate-capped gold nanoparticles into supraparticles with crystallographically aligned building blocks.

## Introduction

The collective oscillations of electrons in the conduction band of gold nanoparticles are responsible for the strong surface electromagnetic fields observed in these nanomaterials.^1^ Assembling these nanoparticles into compact supraparticles with well-defined 3D structures allows for engineering their plasmon resonances and also intensifies the electromagnetic field at nanoparticle interstices.^2^ This phenomenon makes gold supraparticles extremely promising materials for nanomedicine applications such as photothermal therapy and *in vivo* sensing. For example, in photothermal therapy closely packed nanoparticles require less energy to generate plasmonic heat, which minimizes side effects originating from the incident light.^3^ The strong electromagnetic fields found in strongly coupled supraparticles can be used to boost the signal of surface enhanced Raman scattering (SERS), which is useful in *in vivo* sensing.^4^ If soluble in water and not cytotoxic, these plasmonic supraparticles can also be used in combined therapeutics and diagnostics (theranostics).^5^

Here, a new method is introduced for assembling closely packed gold supraparticles with crystallographically aligned nano-building blocks. The supraparticles are made of citrate-capped gold nanoparticles and are assembled in a biocompatible matrix in the absence of toxic ligands or organic solvents. Previous approaches for the assembly of closely packed supraparticles required drop casting the nanoparticle building blocks.^6^ Usually this approach yields supraparticles supported on a substrate that would be difficult to re-disperse in aqueous solution for nanomedicine applications. Some of these approaches also require using organic solvents and surfactants that raise serious toxicological concerns for *in vivo* applications.^7^ Similar issues could jeopardize the utilization of covalently linked supraparticles in bioimaging and nanomedicine.^8^ Other approaches for obtaining gold supraparticles rely on modifying nanoparticle building blocks with biomolecules and assembling them through programmed biorecognition reactions.^9^ For example, DNA-decorated nanoparticles can be assembled into exquisite supraparticles with designer superlattices.^10^ Streptavidin-modified nanoparticles yield supraparticles with highly aligned nanoparticle building blocks in the presence of genetically engineered collagen nanowires.^11^ Despite the outstanding degree of control over the supraparticle structure afforded by these approaches, the nanoparticle building blocks are separated several nanometers away due to the presence of biomolecular ligands around them, and therefore their surface plasmons are not as strongly coupled as in the drop-casted supraparticles.

The method proposed here overcomes previous limitations for generating compact plasmonic supraparticles dispersed in water suitable for nanomedicine applications. The method is inspired by biomineralization processes that generate supraparticles showing a high degree of crystallographic alignment.^12^ Mineral supraparticles, which are also known as mesocrystals, often grow within a polymer matrix that facilitates the nucleation, growth and oriented attachment of nanoparticle building blocks.^13^ For example it has been shown that CaCO_3_ supraparticles can be grown *in vitro* using a cellulose acetate (CA) scaffold in the presence of poly(acrylic) acid.^14^ An *N*-trimethylammonium derivative of hydroxyethyl cellulose could also grow CaCO_3_ supraparticles *via* aggregation-mediated crystallization.^15^ Motivated by these findings, we induced the self-assembly of citrate-capped nanoparticles by confining them within a CA membrane. The supraparticles obtained with this method have crystallographically aligned and strongly coupled nanoparticle building blocks, which results in enhanced plasmonic properties for SERS and photothermal therapy applications.

## Results and discussion

**Fig. 1:**
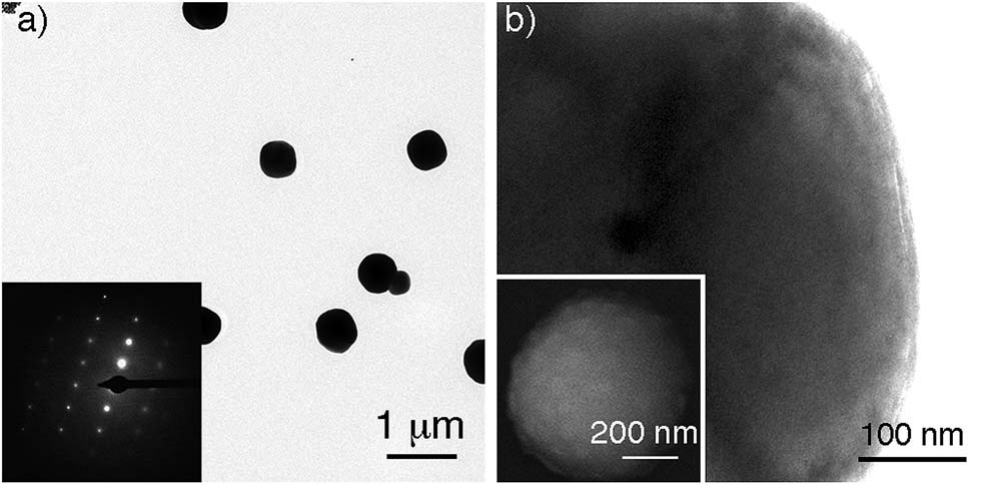
Gold supraparticles found after filtering gold nanoparticles through a CA membrane; (a) TEM images of the supraparticles; inset: SAED pattern of a single supraparticle; (b) high-magnification TEM image of a supraparticle; inset: SEM image of a supraparticle.

Gold supraparticles were assembled by filtering a solution containing citrate-capped nanoparticles through a CA membrane. The process was carried out at room temperature. The nanoparticle building blocks had an average diameter of 40 nm (Fig. S1 in the ESI†). The membranes contain micrometric pores that form a 3D mesh with a cut-off value of 0.2 μm (Fig. S2 in ESI†).^16^ It was found that the membrane saturated easily and acquired the typical red tint of the nanoparticle building blocks (Fig. S3 in ESI†). When the filtered nanoparticles were imaged with transmission electron microscopy (TEM), a new population of larger particles with a diameter of 0.3–0.6 μm was found that was not present before filtering (Fig. 1a and S4 in ESI†). The selected area electron diffraction (SAED) pattern of one of the large particles consists of single spots and demonstrates that the nanoparticles are made of gold (Fig. 1a, inset, and S5 in ESI†). This SAED pattern is characteristic of a single crystal.^17^ Polycrystalline materials yield a mixture of dot patterns or rings originating from the different orientations of their crystalline domains with respect to the incident e-beam.^18^ High-resolution TEM and scanning electron microscopy (SEM) images in Fig. 1b show that the large particles contain rough surfaces and areas of different electron density that match the size of the building blocks. This observation is in agreement with the idea that the large particles are highly compact nanoparticle assemblies made of smaller building blocks.

Two possible hypotheses could justify the results seen in Fig. 1. In the first hypothesis the nanoparticle building blocks are subject to an Ostwald ripening process inside the filter that yields large gold particles with single-crystal SAED patterns. This would require the dissolution of the nanoparticle building blocks followed by their re-growth as larger, single-crystal particles. This scenario is extremely unlikely because the Ostwald ripening of citrate-capped Au nanoparticles happens at high temperatures,^19^ while the filtering process was performed at room temperature (18–22 °C). Furthermore the extinction spectrum of the supraparticles is totally different from the spectrum of large spherical gold nanoparticles^19^ or platonic gold nanocrystals (Fig. S8 in ESI†).^20^ The second hypothesis consists in the assembly of a mesocrystal. Mesocrystals are supraparticles containing nanocrystals with common crystallographic order.^12^ Mesocrystals can be identified by their single-crystal SAED patterns.^12^ The supraparticles shown in Fig. 1 are made of smaller nanoparticle building blocks and have single-crystal SAED patterns (Fig. 1b). Therefore we propose that they are mesocrystals made of crystallographically aligned nanoparticle building blocks.^12^

**Fig. 2:**
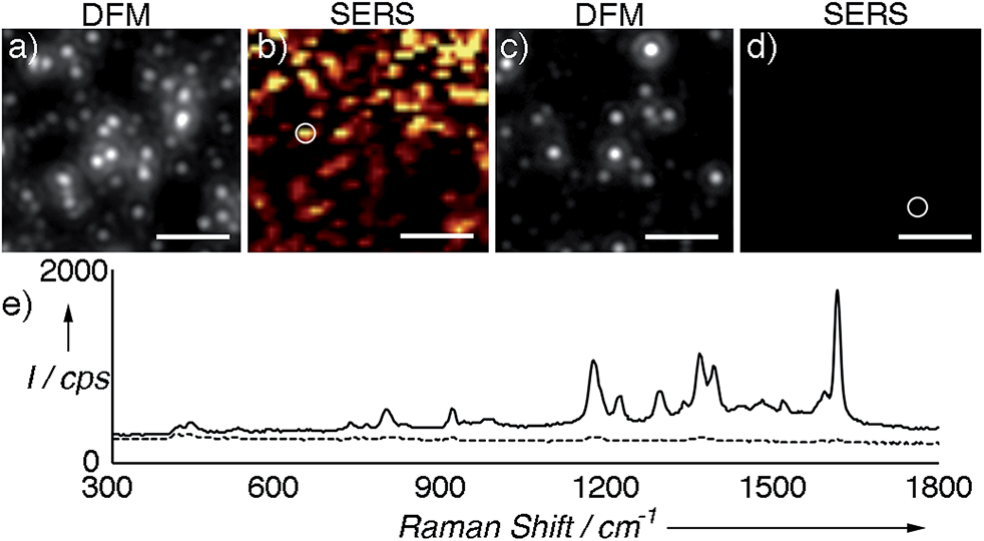
SERS analysis of supraparticles modified with malachite green isothiocyanate; (a) and (b) correlated dark-field microscopy (DFM) image and SERS map of supraparticles adsorbed onto a glass slide; (c) and (d) DFM image and SERS map of nanoparticle building blocks; (e) representative SERS spectra of supraparticles (solid line, highlighted with a circle in (b)) or nanoparticle building blocks (dotted line, circle in (d)). SERS images were generated from analysis of the peak intensity at ∼1172 cm^–1^ with respect to the background signal with the brightest points corresponding to intensities >300 counts per s (cps). See also Fig. S7 in ESI† and experimental details below. Scale bars: 5 μm.

Next we compared the SERS signals generated by either the supraparticles or the nanoparticle building blocks. The colloids were modified with a Raman reporter (malachite green isothiocyanate, Fig. S6 in ESI†) and immobilized on a glass slide modified with a positively charged polymer (PDDA). Great care was taken to not leave the samples to dry on the slide in order to avoid the formation of uncontrolled aggregates. Fig. 2a and b show a dark-field microscopy (DFM) image and a SERS map of an area containing supraparticles, respectively (see also Fig. S7 in ESI†). A representative SERS spectrum is also provided in Fig. 2e. Fig. 2c and d show a DFM image and SERS map of an area containing individual nanoparticle building blocks. From these images and spectra, it is evident that the supraparticles generate SERS signals that are several orders of magnitude higher than the individual nanoparticle building blocks. This large increase in the SERS signal agrees well with the formation of plasmonic hot spots,^2^ and therefore demonstrates that the nanoparticle building bocks are in close contact in the supraparticles. Together, the results shown in Fig. 1 and 2 indicate that the supraparticles are mesocrystals containing crystallographically aligned and strongly coupled nanoparticle building blocks.

**Fig. 3:**
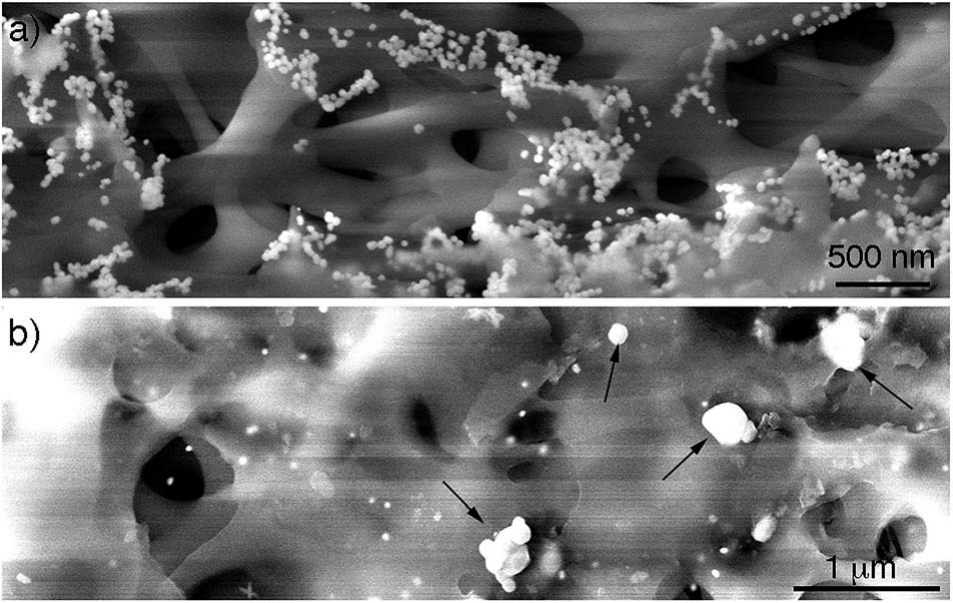
SEM images of CA membranes; (a) membrane top showing nanoparticle building blocks interacting with the membrane; (b) membrane bottom showing large electron-dense particles (indicated by arrows).

After demonstrating that the large particles found after filtering are nanoparticle assemblies and not single particles, the mechanism of assembly of the supraparticles was studied. To this end, the CA membrane was imaged after filtering the gold nanoparticles. Fig. 3a shows a SEM image of the top of the membrane. In this image it can be seen that the nanoparticles attach to the membrane, which is in agreement with the observation of a residual red tint after filtering in Fig. S3.† These results are also in line with previous publications reporting the interaction between citrate-capped gold nanoparticles and cellulose *via* van der Waals interactions.^21^ Moreover, nanoparticles covered with carboxylated polyethylene glycol ligands, which are also negatively charged, did not attach to the filter, and polyethersulfone (PES) filters with a similar cut-off yet different chemical composition did not accumulate citrate-capped nanoparticles, which further demonstrates that interactions between citrate and CA are responsible for the attachment of nanoparticles to the membrane (Fig. S3 in ESI†). Furthermore, no supraparticles were detected in samples filtered through the PES membrane (Fig. S9 in ESI†), which also indicates that interactions between the CA and the gold nanoparticles are essential for obtaining supraparticles. In Fig. 3a it can also be observed that the nanoparticles form aggregates on the membranes. However, these aggregates are not present at the bottom of the membrane (Fig. 3b). In this region, large particles are found along with non-aggregated nanoparticle building blocks. The large particles are electron-dense and have a morphology similar to that of the supraparticles in Fig. 1, which strongly suggests that they are supraparticles assembled at the bottom of the membrane.

**Fig. 4:**
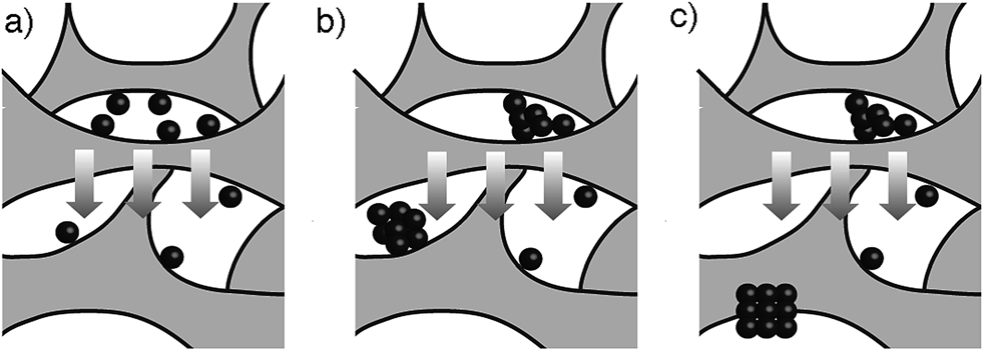
Schematic representation of the main steps involved in the formation of gold supraparticles upon filtration through a CA membrane; (a) gold nanoparticles interact with the CA membrane and reduce the pore size; (b) nanoparticle aggregates are formed; (c) as they flow through the filter the nanoparticles in the aggregates realign and generate crystallographically aligned supraparticles at the bottom of the filter. The arrows indicate the direction of the solution flow.

In view of the abovementioned observations, a proposed mechanism for the assembly of gold supraparticles is schematized in Fig. 4. When the nanoparticles are filtered through the CA membrane some attach to the surface of the filter (Fig. 3a, 4a and S3†). This reduces the pore size and the flow rate through the filter, therefore increasing the local concentration of the nanoparticles around the pores. The increased concentration leads to the formation of aggregates (Fig. 3a and 4b), which further reduce the pore size and the flow rate through the membrane. Saturation of the filter with nanoparticles is a crucial step towards the formation of supraparticles, since smaller 20 nm diameter citrate-capped nanoparticles did not saturate the filter and did not generate supraparticles (Fig. S10 in ESI†). As the solution is filtered through the membrane some gold aggregates are pushed to the bottom of the filter (Fig. 4b). During this process the nanoparticles in the aggregates reorganize and align their crystal lattices to form mesocrystals, that is, supraparticles with SAED patterns similar to those of a single crystal (Fig. 1a and 4c).^12^ The nanoparticle building blocks are not perfectly spherical and show a polydisperse size distribution. This means that the assembly of the supraparticles involved a self-selecting process of nanoparticle building blocks, which is in line with previous reports on the assembly of supraparticles containing shaped nanocrystals.^22^ Since the building blocks are shaped, interactions between parallel surface facets and the resultant increase of excluded volume between nanocrystals must play a significant role in the assembly of nanocrystals.^23^ In Fig. 3 and S4† some nanoparticles are found assembled as chain-like structures, which suggests that dipolar interactions may be involved in the mechanism of self-assembly.^29^ It has also been proposed that the oriented attachment of gold nanoparticles requires ligand loss followed by lattice alignment.^24^ SERS signals of the citrate-capping ligands are weaker in the supraparticles compared to a solution containing the same concentration of non-aggregated nanoparticle building blocks, which suggests that the supraparticles are assembled following a similar ligand displacement mechanism (Fig. S11 in ESI†).

**Fig. 5:**
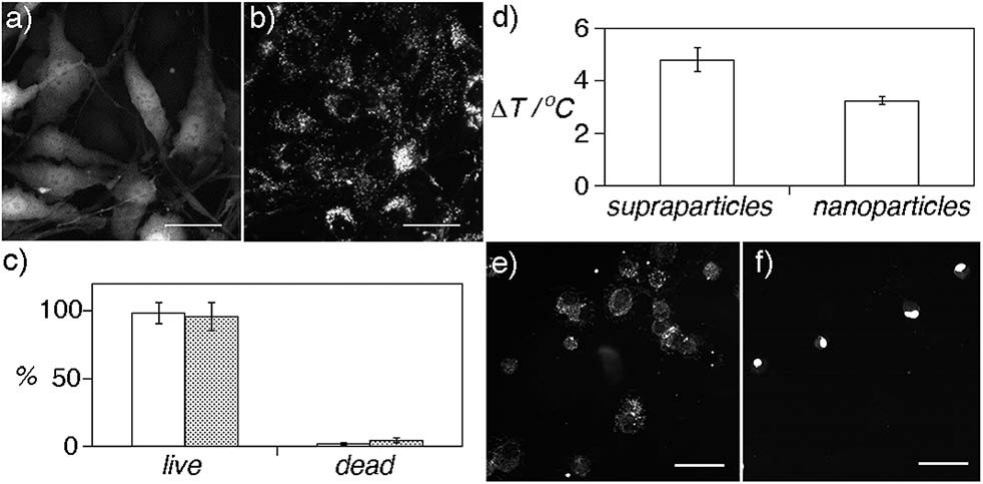
Cell internalization and photothermal effects of gold supraparticles; (a) and (b) correlated fluorescence and dark-field microscopy (DFM) images of GFP-expressing bone cancer cells after adding supraparticles; (c) cytotoxicity of supraparticles (white bars) and nanoparticles (dotted bars); (d) increase in temperature measured after irradiating 20 μL of supraparticles or nanoparticle building blocks for 1 min with the 515 nm laser. The laser power incident onto the sample was 27 mW; (e) and (f) correlated DFM and fluorescence images of prostate cancer cells after irradiation with a 515 nm laser for 15 min and staining with ethidium bromide, fluorescent cells in (f) are dead; (f) scale bars: 50 μm. Error bars are the standard deviation (*n* ≥ 3).

The highly coupled supraparticles obtained here are assembled in a biocompatible CA matrix, have sub-micrometer sizes, and are dispersed in aqueous solution, which makes them promising candidates for applications in nanomedicine. Before exploring these applications, the potential cytotoxicity of the supraparticles was studied (Fig. 5a–c). Fig. 5a and b show fluorescence and DFM images of bone cancer cells incubated with supraparticles. The cells are fluorescent because they express green fluorescent protein (GFP). After washing the cells three times, the supraparticles were found only in the cell cytoplasm (Fig. 5b) (see also Fig. S12 in ESI†). Staining with trypan blue revealed that the cells were alive 36 h after internalizing nanoparticles or supraparticles, which demonstrates that they are highly biocompatible (Fig. 5c).

Next, we studied the photothermal properties of supraparticles and nanoparticle building blocks. Both nanoheaters absorb green light. Therefore they were excited with a partially focused CW laser at 515 nm in order to discern whether the assembly of the supraparticles results in improved photothermal properties compared to the nanoparticle building blocks. Irradiating plasmonic nanoheaters results in a linear increase of the temperature of the solution with time followed by a plateau where the temperature remains almost constant with time.^25^ Consequently the samples were irradiated for only 1 min in order to compare the generation of heat by supraparticles and nanoparticle building blocks when the temperature changes linearly with time, since in this region the comparison is more accurate. In Fig. 5d, the increase in temperature measured in the solution containing supraparticles is 50% higher, which demonstrates that the proposed supraparticles generate more plasmonic heat than the nanoparticle building blocks when excited with a laser than can be absorbed by both nanostructures and at the same nanoparticle concentration. These results are in line with previous studies that show that nanoparticle assemblies generate more heat than spherical or shaped nanoparticles.^26^

After comparing the generation of heat by supraparticles and nanoparticle building blocks their ability to kill cancer cells *via* photothermal effects was studied. In these experiments the irradiation time was increased to 15 min so that the nanoheaters could reach a temperature high enough to kill the cells. First, a solution containing concentrated prostate cancer cells and supraparticles was irradiated with the 515 nm laser. After 15 minutes, 13 ± 3% of the cells were found to be dead in the solution. Cell death was corroborated by adding ethidium bromide, which becomes fluorescent upon interacting with the DNA in the nucleus of dead cells (Fig. 5e and f) (see also Fig. S13 in ESI†). When the same experiment was repeated without supraparticles only 5 ± 1% of the cells were dead, therefore demonstrating that cell death was induced by plasmonic heat and not by the laser. Furthermore, when the cells were incubated with nanoparticle building blocks at the same concentration, 30% less cells were dead in the solution (9 ± 1% dead cells). These results demonstrate that supraparticles are able to kill cancer cells more efficiently than nanoparticle building blocks when excited with a source of light that can be absorbed by both types of nanoheaters. Although the 515 nm laser is used here to compare the photothermal properties of nanoparticle building blocks and supraparticles, in real nanomedicine applications the broadband absorption of the supraparticles would allow exciting them with near-infrared lasers, which enable a deeper tissue penetration depth without overheating the irradiated area (Fig. S14 in ESI†).^27^

## Conclusions

In conclusion, we have demonstrated that citrate-capped gold nanoparticles can be assembled into supraparticles when filtered through a CA membrane. The supraparticles diffract like a single crystal, which demonstrates that the constituent building blocks are crystallographically aligned. Furthermore, the supraparticles have nanoparticle building blocks in close contact that generate highly intense SERS signals. They also generate plasmonic heat more efficiently and kill more cancer cells than the constituent nanoparticle building blocks. These traits make the proposed crystallographically aligned supraparticles promising candidates for nanomedicine applications such as SERS-based diagnostics,^4^ photothermal therapy^3^ and plasmonics-based theranostics.^5^

## Experimental

### Synthesis of nanoparticle building blocks and assembly of gold supraparticles

Citrate-capped nanoparticles were obtained by adding 57.5 mg of sodium citrate dissolved in 7.5 mL of water to 500 mL of boiling water containing 60.5 mg of sodium tetrachloroaurate under continuous stirring. The mixture was boiled for 15 minutes. This method yields citrate-capped gold nanoparticles.^28^ The nanoparticle solution was left at room temperature for several days before assembling supraparticles. To obtain supraparticles, 40 mL of the nanoparticle solution was filtered with a syringe through a cellulose acetate membrane (0.2 μm cut-off, VWR) at room temperature (18–22 °C).

### Electron microscopy imaging

1–2 μL of sample was let to dry on a carbon grid. TEM imaging was performed with a FEI Tecnai T20 TEM operating at an acceleration voltage of 200 kV. SEM imaging was performed with a FEI Quanta 250 FEG-ESEM. 20 mL of water was filtered through the membranes prior to SEM imaging in order to remove any salts and loosely bound nanoparticles.

### Dark field microscopy (DFM) and SERS experiments

Glass slides were cleaned by ultrasonic treatment in acetone for 5 min followed by rinsing with ethanol and deionized water, and drying with nitrogen. The slides were then immersed in Hellmanex™ for at least 1 h, rinsed with abundant water and dried with nitrogen. Subsequently the slides were immersed in a 1% (v/v) solution of poly(diallyldimethylammonium chloride) (PDDA) for 30 min. The slides were then rinsed with deionized water and dried with nitrogen. 100 μL of nanoparticle solution (either containing supraparticles or a suspension of the smaller nanoparticle building blocks) was placed onto the substrates for 10 min. The nanoparticles were adsorbed onto the PPDA-covered slide due to the electrostatic interaction between the citrate-capped nanoparticles and positively charged glass surface. After 10 min the slides were rinsed with water first and then dried with nitrogen. This prevents the formation of drying-induced aggregates being present on the slide surface. The slides were then covered with 1 μM malachite green solution (diluted from a 1 mM stock solution in ethanol) for 10 minutes, rinsed with water and dried with nitrogen.

Correlated dark-field and SERS imaging was performed on two different microscopes with reference marks on the slide surface used to identify different regions. Raman maps were obtained using a confocal WITec Alpha300R instrument at 633 nm excitation. All maps were acquired using a 100× objective (Olympus MPlan, NA = 0.9). Areas up to 20 × 20 μm in size were imaged in ∼0.4 μM steps. An incident laser power of ∼0.9 mW and signal integration time of 1 s was used throughout. The SERS maps were created by plotting the difference between the maximum and minimum intensities in the 1020–1185 cm^–1^ window, targeting the peak at 1172 cm^–1^ and a preceding background region of the spectrum. The same image acquisition conditions were used (*e.g.* incident light intensity, integration time) for both the supraparticle and control slides when performing the SERS measurements to enable a direct comparison of relative Raman intensities.

Dark-field images were acquired using a Nikon Eclipse LV100 with a 50× objective (Nikon CFI LU Plan BD ELWD, NA = 0.55) in an epi dark-field configuration. Images were acquired using a Coolsnap camera and using the same source light intensity and exposure times.

### Cell culture and supraparticle/nanoparticle internalization

The nanoparticles and supraparticles were modified with thiolated PEG (MW = 5000) in order to avoid their aggregation in cell media. Cells were incubated with nanoparticles or supraparticles at the same nanoparticle concentration (same absorbance at 400 nm). Prostatic small cell carcinoma (PC3) was kindly gifted from Professor Duncan Graham at the department of Pure and Applied Chemistry, University of Strathclyde, UK. GFP-expressing bone cancer MG-63 was kindly gifted from Dr. Eileen Gentleman, King's College London, UK. Both cell lines were grown in Dulbecco's modified Eagle's medium supplemented with 10% Fetal Bovine Serum (FBS). Cells were maintained at 37 °C in a 5% CO_2_ humidified environment. Cells were trypsinized and placed on glass slides or Petri dishes two days prior to imaging or photothermal experiments. For imaging experiments, cells were incubated with nanoparticles or supraparticles 36 hours (10 μL in 2 mL of medium). Incubations were performed in medium supplemented with 0.05% FBS. On the day of the experiment, the medium was removed and replaced with new medium. Cytotoxicity and cell death assays were performed using trypan blue or ethidium bromide following common protocols described elsewhere. Dark-field and fluorescent images were obtained with a Nikon Eclipse LV100 with a 20× objective.

### Photothermal experiments

The day of the experiments, PC3 cancer cells were trypsinized and centrifugated 3 times for 5 minutes at 1000 rpm. After the last centrifugation, the supernatant was removed and cells were re-suspended in 50 μL of PBS. 2 μL of nanoparticles or supraparticles were added to the cell solution before irradiation (2 μL of PBS was added to the control cells). A green continuous wave He-ion laser (515 nm, 27 mW at the focus point) was used to irradiate the different samples for 15 minutes. To measure the increase in temperature generated by plasmonic heat, 20 μL of nanoparticles or supraparticles were irradiated with the laser for 1 minute. The temperature in the drop was measured with a thermocouple (Digital Meter, model 6802 II).

## Supplementary Material

Supplementary information
