# Supplementary material for: Self-assembly of gold supraparticles with crystallographically aligned and strongly coupled nanoparticle building blocks for SERS and photothermal therapy
Source: Chem Sci. 2016 Jun 20;7(9):6232–7. doi: 10.1039/c6sc02465c (PMC6024203; doi:10.1039/c6sc02465c)
Supplement: Supplementary file 1 [file SC-007-C6SC02465C-s001.pdf]

Electronic Supplementary Material (ESI) for Chemical Science.
This journal is © The Royal Society of Chemistry 2016

Supporting Information for:

Self-Assembly of Gold Supraparticles with Crystallographically Aligned and Strongly Coupled Nanoparticle Building Blocks for SERS and Photothermal Therapy

Sureyya Paterson,[a] Sebastian A. Thompson, [b] Jennifer Gracie,[a] Alastair W. Wark,[a] and Roberto de la Rica [a],*

[a]WestCHEM, Department of Pure and Applied Chemistry, University of Strathclyde, Technology and Innovation Centre, 99 George Street, Glasgow, G1 1RD, Scotland, UK

[b]Department of Chemistry and Biochemistry, Hunter College - City University of New York, New York 10065, USA

Contents:

Figure S1. TEM image of the citrate-capped nanoparticle building blocks.

Figure S2. SEM image of the CA membrane.

Figure S3. Images of different membranes after filtering nanoparticles.

Figure S4. Additional TEM images and size distribution of gold supraparticles.

Figure S5. SAED of a single supraparticle.

Figure S6. Raman spectrum of malachite green isothiocyanate.

Figure S7. Dark field microscopy (DFM) and SERS maps of supraparticles.

Figure S8. Extinction spectra of nanoparticles and supraparticles.

Figure S9. 40 nm-diameter nanoparticles after filtering through the PES membrane.

Figure S10. 20 nm-diameter nanoparticles after filtering through the CA membrane.

Figure S11. SERS spectra of citrate-capped gold nanoparticles.

Figure S12. Cancer cells incubated with supraparticles.

Figure S13. Images of cancer cells after irradiating them with a 515 nm laser.

Figure S14. Heat generation of supraparticles irradiated with 785 nm CW laser

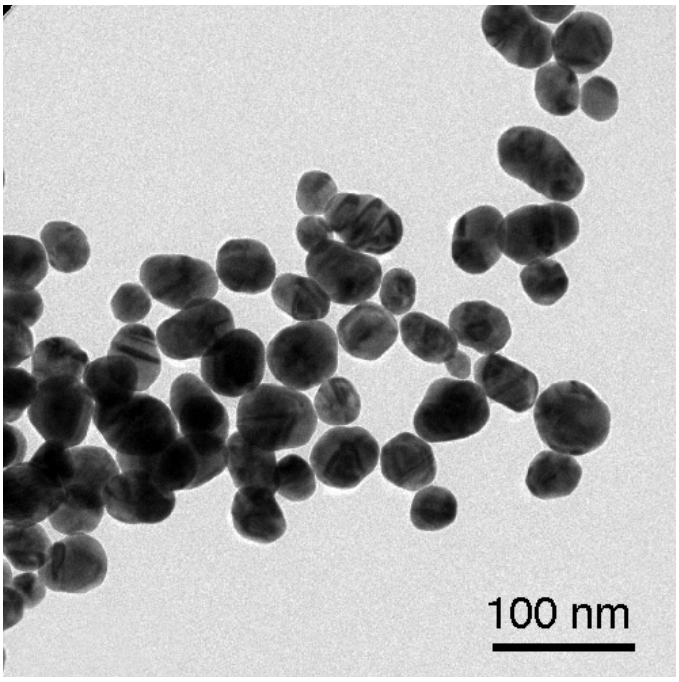

Figure S1. TEM image of the citrate-capped nanoparticle building blocks.

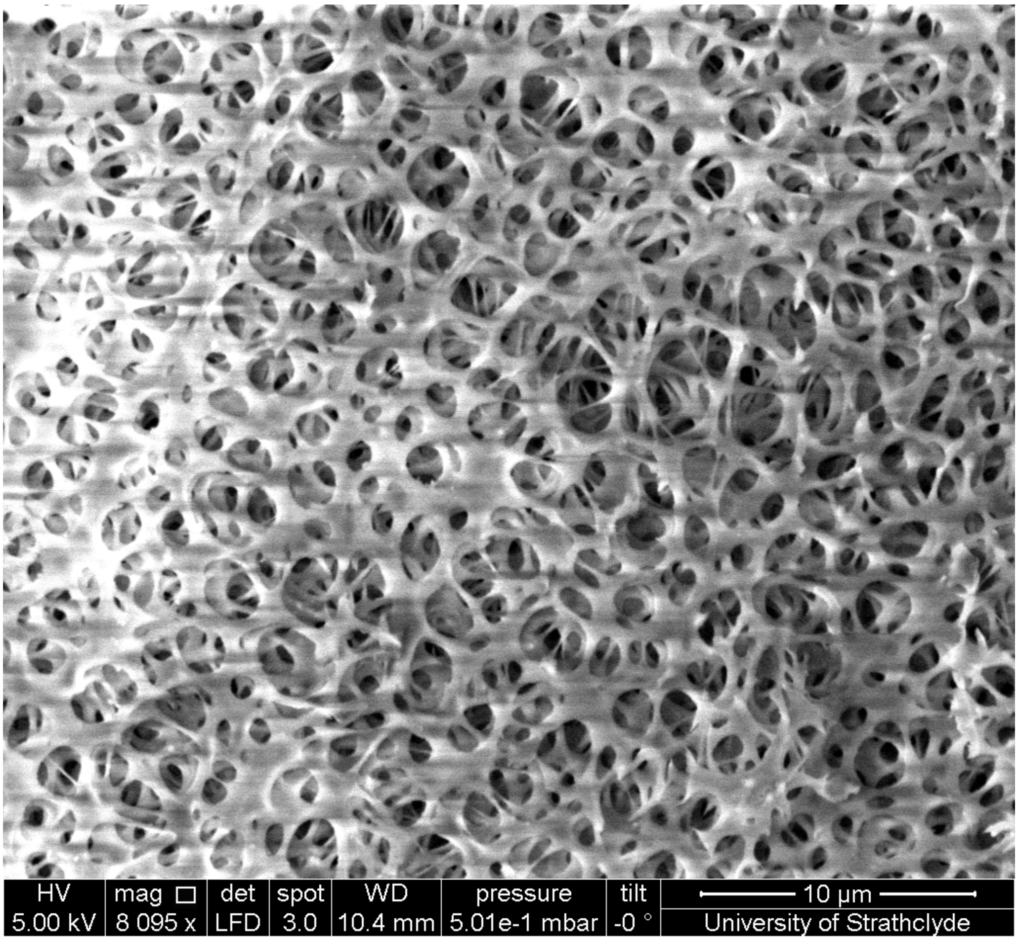

Figure S2. SEM image of the CA membrane.

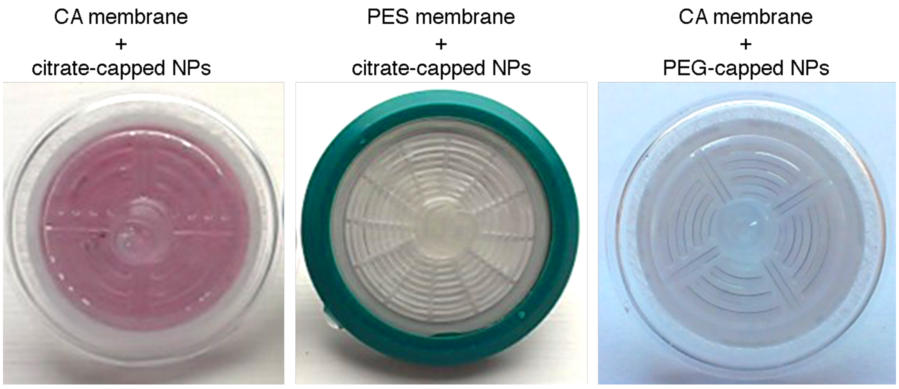

Figure S3. Images of different membranes after filtering citrate-capped nanoparticles or nanoparticles decorated with thiolated PEG. CA: cellulose acetate, PES: polyethersulfone. The images were taken after filtering 40 mL of nanoparticles followed by filtering 20 mL of deionized water. Both filters have a cut-off of 0.2 μm. The PEG-capped nanoparticles were obtained by adding 0.1 mM carboxy-PEG_{12}-thiol (ThermoFisher Scientific) to the citrate-capped nanoparticles overnight.

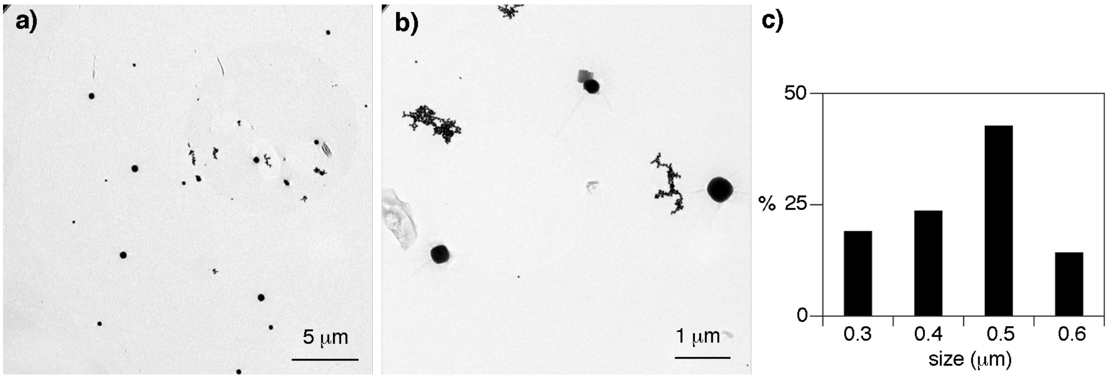

Figure S4. a), b) Additional TEM images of gold supraparticles and c) size distribution plot.

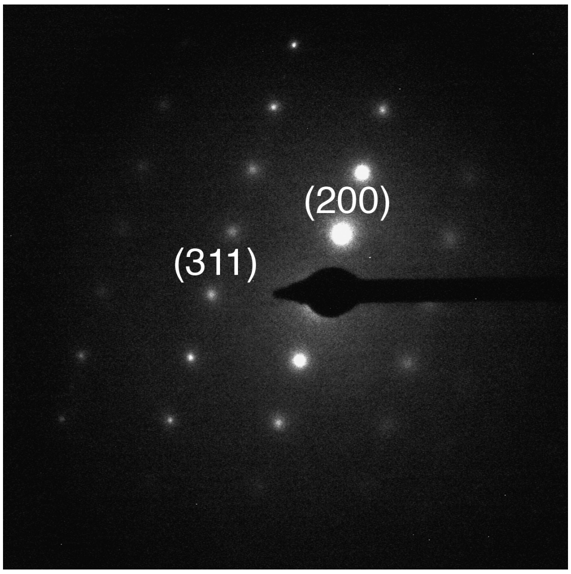

Figure S5. SAED of a single supraparticle.

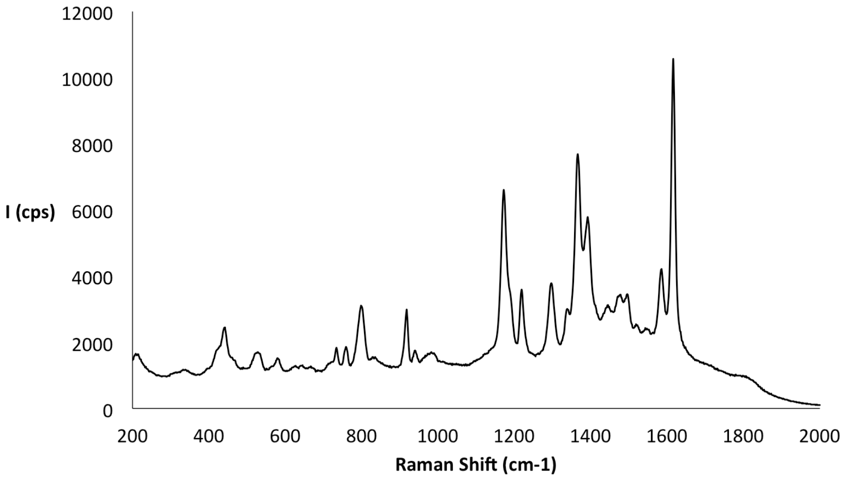

Figure S6. Bulk Raman spectrum of a solution of 40 nm gold nanoparticle building blocks modified with 1 μM malachite green isothiocyanate, obtained with a Snowy Range Instrument (model Sierra 2.0, excitation wavelength 638 nm).

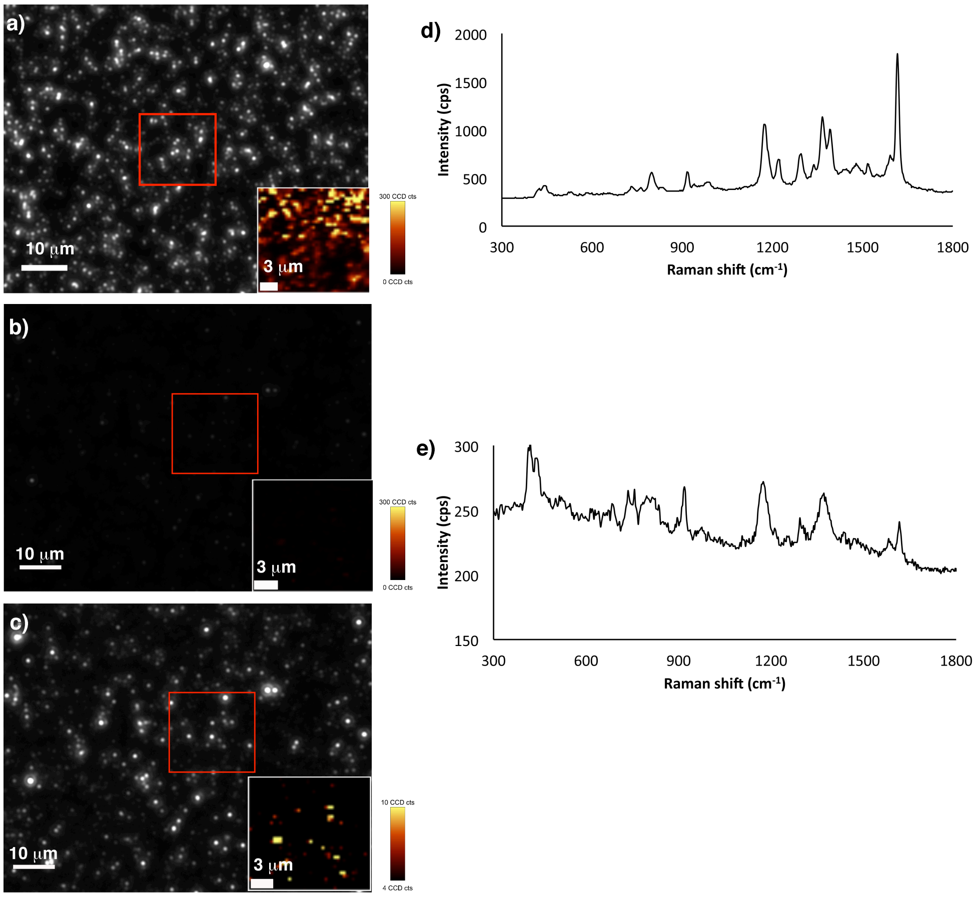

Figure S7. Dark field microscopy (DFM) and SERS maps (insets taken from red square areas) of a) sample containing supraparticles; b) and c) sample containing nanoparticle building blocks; d) and e) show a representative SERS spectrum of a) and b), respectively. DFM and SERS images in b) were taken in the same conditions as in a); the contrast of these images was not altered. In (a), the lowering of SERS intensities from top to bottom of the image is due to a small focus drift during the mapping process. Lower scattering intensities are observed in DFM images in b) compared to a) because the nanoparticle building blocks are smaller. In c) the integration was increased from 0.15 to 0.75 s in DFM images and the contrast of the SERS image was enhanced (lower Z scale). This enabled visualizing the smaller and less SERS active nanoparticle building blocks. SERS signal in (a) and (d) are orders of magnitude larger than in (b) and (e), which is in agreement with the presence of hot spots in the supraparticles. It has been reported that supraparticles with sharp edges may show increased SERS enhancements at their corners.[2] This can only increase SERS signals up to 2 times.[2] The supraparticles proposed here have rounded edges (Fig. 1) and show SERS enhancements several orders of magnitude higher than the individual nanoparticle building blocks. Therefore any changes in their SERS properties derived from the overall shape of the supraparticles are irrelevant compared to the formation of hot spots in the superstructure.

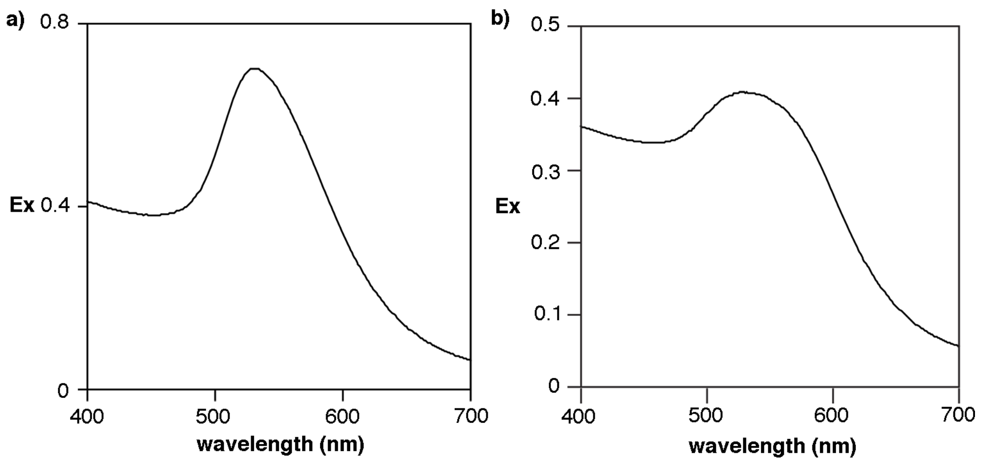

Figure S8. Extinction spectra of (a) nanoparticle building blocks before filtering and (b) gold supraparticles. Supraparticles were obtained from the filtrate by centrifuging for 3 min at 1000 rpm, removing the supernatant, and dispersing the supraparticles in water.

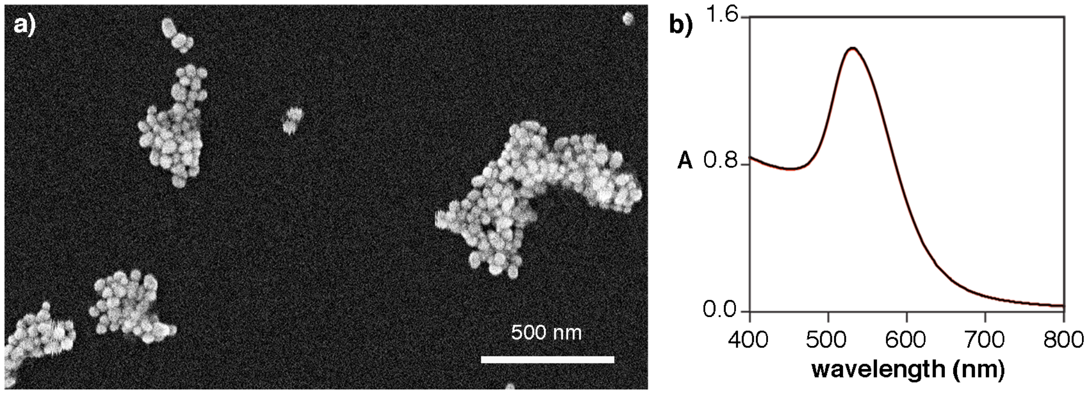

Figure S9. 40 nm-diameter citrate-capped gold nanoparticles after filtering through the PES membrane; (a) Only unorganized aggregates are observed, which are generated when drying the sample on the carbon grid. This is shown in the UV-Vis spectrum of the nanoparticle solution in (b), which does not change after filtering the nanoparticles (black: before filtering, red: after filtering).

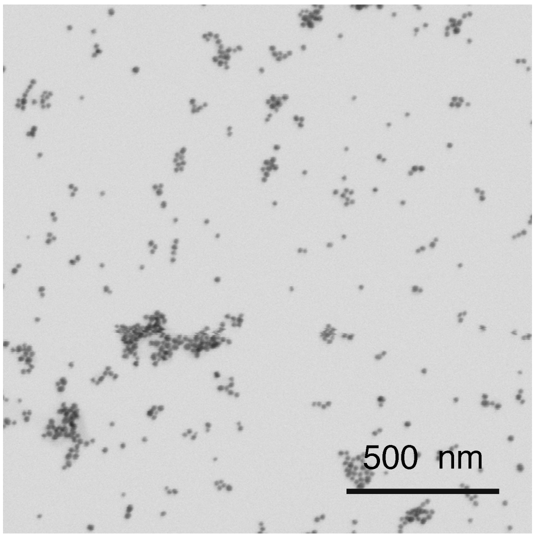

Figure S10. Electron microscopy image of 20 nm-diameter citrate-capped gold nanoparticles after filtering through the CA membrane. Only unorganized aggregates are observed, which are generated when drying the sample on the carbon grid.

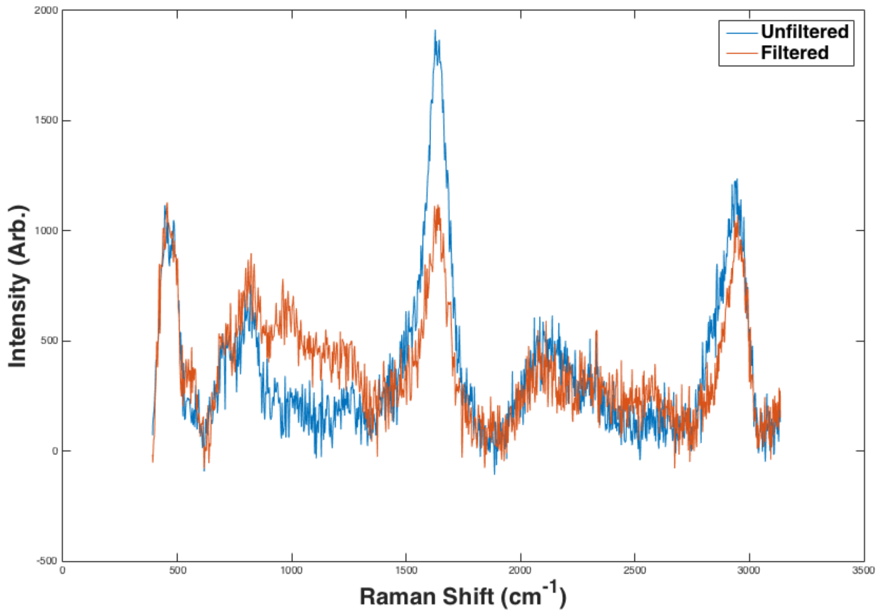

Figure S11. Bulk solution SERS spectra of citrate-capped gold nanoparticles before (blue) and after (orange) filtering through the CA membrane. Both samples had the same nanoparticle concentration estimated from the absorbance at 400 nm. The spectra were obtained with a Snowy Range instrument (model Sierra 2.0, excitation wavelength 532 nm). The peak at 1627 cm^{-1} is characteristic of citrate[1]. There is a lower SERS citrate background signal in the sample containing supraparticles despite the presence of hotspots, which indicates that there is a reduced number of citrate capping ligands around the supraparticles.

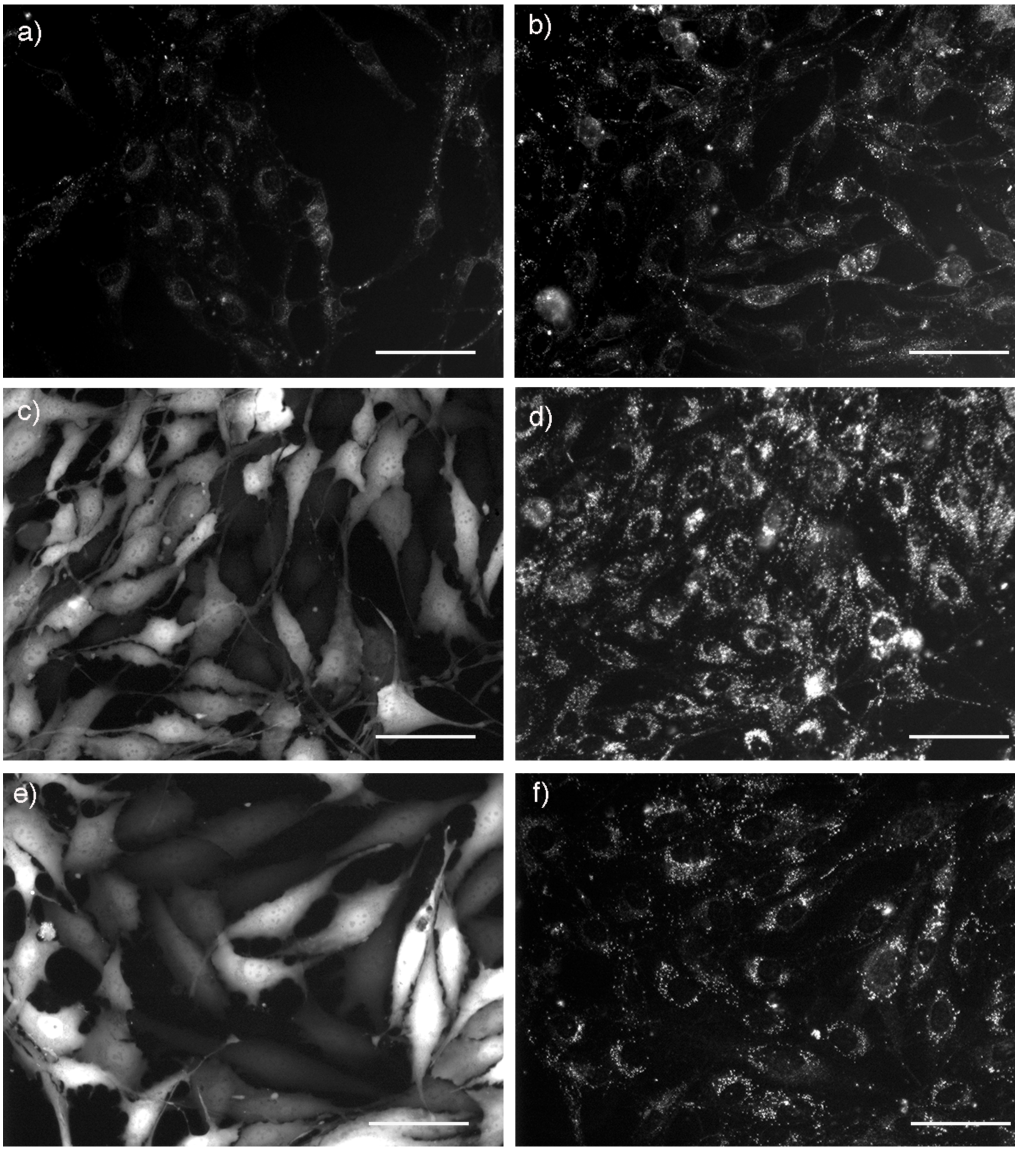

Figure S12. Cancer cells incubated with supraparticles; In-situ dark-field microscopy (DFM) images of prostate cancer cells, a) without supraparticles; b) with supraparticles; the increased contrast in b) compared to a) indicates that the supraparticles were internalized by the cells, the supraparticles are localized in the cytoplasm; c) fluorescence and d) DFM images of bone cancer cells bearing gold supraparticles; e) fluorescence and f) DFM images of bone cancer cells not bearing gold supraparticles (not incubated with supraparticles). The cells are fluorescent because they have been genetically engineered to express green fluorescent protein. Nanoparticles in (d) are localized in the cytoplasm of the cells. Scale bars: 100 μm.

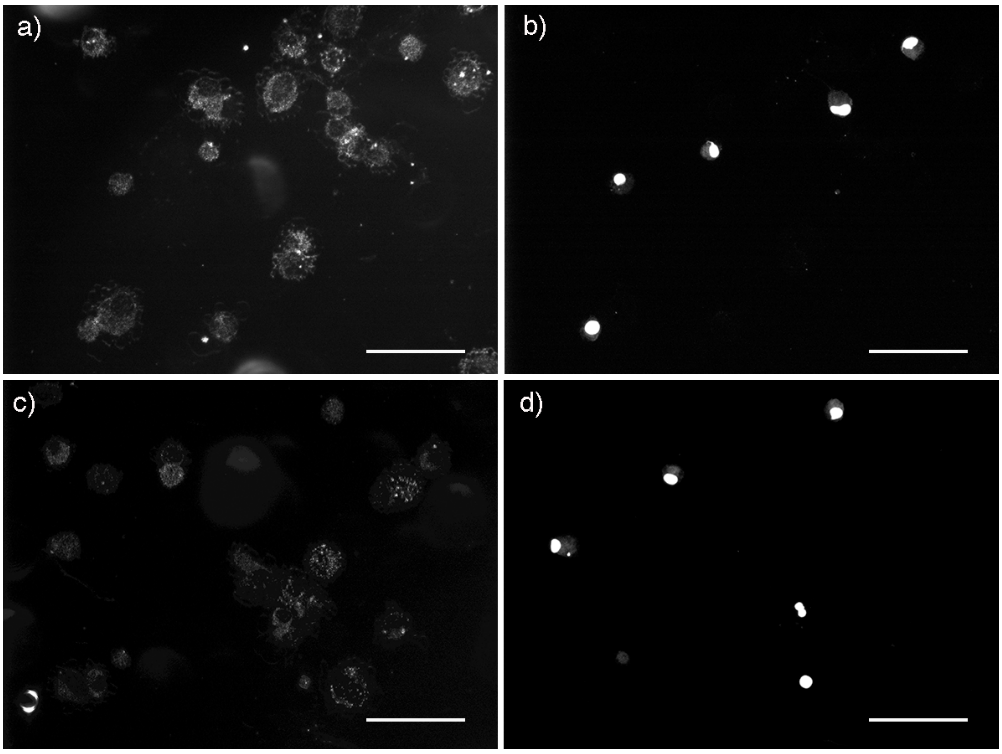

Figure S13. In-situ images of prostate cancer cells incubated with supraparticles after irradiating them in solution with a 515 nm laser for 15 minutes; a) c) dark-field microscopy images; b) and d) correlated fluorescence microscopy images. Ethidium bromide was used to stain the nuclei only in dead cells. Cells with fluorescently stained nuclei in b) and d) are dead. Scale bars: 100 μm.

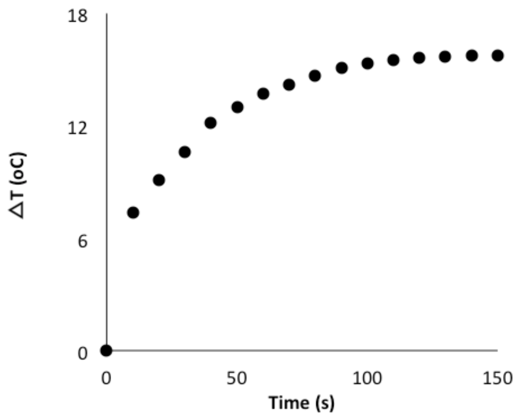

Figure S14. Increase in temperature generated by supraparticles irradiated with a 785 nm CW laser (235 mW at the point of incidence) as a function of irradiation time.

References

[1] Y. Zhang, F. Wang, H. Yin, Min Hong, Advances in Nanoparticles **2013**, *2*, 104.

[2] Z. Zhu, H. Meng, W. Liu, X. Liu, J. Gong, X. Qiu, L. Jiang, D. Wang and Z. Tang, Angew. Chem. Int. Ed., **2011**, 50, 1593.
